# Interaction between Shiga Toxin and Monoclonal Antibodies: Binding Characteristics and *in Vitro* Neutralizing Abilities

**DOI:** 10.3390/toxins4090729

**Published:** 2012-09-18

**Authors:** Letícia B. Rocha, Daniela E. Luz, Claudia T. P. Moraes, Andressa Caravelli, Irene Fernandes, Beatriz E. C. Guth, Denise S. P. Q. Horton, Roxane M. F. Piazza

**Affiliations:** 1 Bacteriology Laboratory, Butantan Institute, São Paulo, SP, Brazil; Email: leticiarocha@butantan.gov.br (L.B.R.); daniedaluz@butantan.gov.br (D.E.L.); claudiatrigo@butantan.gov.br (C.T.P.M.); andressacaravelli@butantan.gov.br (A.C.); dhorton@butantan.gov.br (D.S.P.Q.H.); 2 Immunopathology Laboratory, Butantan Institute, São Paulo, SP, Brazil; Email: irenefernandes@butantan.gov.br; 3 Departament of Microbiology, Immunology, Parasitology, Escola Paulista de Medicina, Federal University of São Paulo, São Paulo, SP, Brazil; Email: bec.guth@unifesp.br.

**Keywords:** Stx1, Stx2, monoclonal antibodies, binding, stability, detection, neutralizing ability, specificity

## Abstract

Monoclonal antibodies (MAbs) have been employed either for diagnosis or treatment of infections caused by different pathogens. Specifically for Shiga toxin-producing *Escherichia coli* (STEC), numerous immunoassays have been developed for STEC diagnosis, showing variability in sensitivity and specificity when evaluated by reference laboratories, and no therapy or vaccines are currently approved. Thus, the aim of this work was the characterization of the interaction between MAbs against Stx1 and Stx2 toxins and their neutralizing abilities to enable their use as tools for diagnosis and therapy. The selected clones designated 3E2 (anti-Stx1) and 2E11 (anti-Stx2) were classified as IgG1. 3E2 recognized the B subunit of Stx1 with an affinity constant of 2.5 × 10^−10^ M, detected as little as 6.2 ng of Stx1 and was stable up to 50 ºC. In contrast, 2E11 recognized the A subunit of Stx2, was stable up to 70 ºC, had a high dissociation constant of 6.1 × 10^−10^ M, and detected as little as 12.5 ng of Stx2. Neutralization tests showed that 160 ng of 3E2 MAb inhibited 80% of Stx1 activity and 500 µg 2E11 MAb were required for 60% inhibition of Stx2 activity. These MAb amounts reversed 25 to 80% of the cytotoxicity triggered by different STEC isolates. In conclusion, these MAbs show suitable characteristics for their use in STEC diagnosis and encourage future studies to investigate their protective efficacy.

## 1. Introduction

Shiga toxin-producing *Escherichia coli* (STEC) strains and their subset, the enterohemorrhagic *E. coli* (EHEC) strains, contain a large pathogenicity island called the locus of enterocyte effacement and carry a 90-kb plasmid [1,2,3]. Not only the O157:H7 serotype, but also some other STEC serotypes have been associated mainly with food-linked outbreaks of Stx-mediated disease with the possibility of a complication such as the hemolytic uremic syndrome (HUS), which is characterized by hemolytic anemia, thrombocytopenia, and renal failure.

Shiga toxins (Stxs) are known to act systemically and therefore must cross from the site of STEC colonization in the gastrointestinal tract to the circulatory system [3]. There are two main subtypes of Stxs, Stx/Stx1 and Stx2. Stx and Stx1 are practically identical, with only one amino acid difference in the A subunit. The A and B subunits of Stx1 and Stx2 differ at the amino acid level by 32 and 27%, respectively, although their crystal structures show high similarity [4,5].

Stxs have the AB_5_ structure, where the active domain (A–32 kDa) contains an *N*-glycosidase. This enzyme depurinates the 28S rRNA of the 60S ribosomal subunit and irreversibly inhibits protein synthesis, resulting in cell death. Subunit B consists of five identical 7.7-kDa monomers that form a pentamer, which allows the C terminus domain of the A2 peptide to traverse it. The B pentamer binds to the eukaryotic receptor globotriaosylceramide (Gb3Cer/CD77) or globotetraosylceramide (Gb4Cer) in the case of Stx2e [6,7,8,9,10], present on the plasma membrane of enterocytes and other cells, for example glomerular endothelial cells [10].

The Vero cell toxicity test detects functionally active toxin and is often used as the gold standard to evaluate diagnostic immunoassays [11,12]. Among the several commercially available immunoassays using monoclonal and/or polyclonal antibodies are VTEC-Screen (Seiken, Japan), Premier EHEC (Meridian Bioscience, US), Ridascreen Verotoxin (R-Biopharm, Germany), ProSpecT Shiga Toxin (Alexon-Trend, US), Immunocard STAT! EHEC (Meridian Diagnostics, US) and Duopath Verotoxins Gold Labeled Immunosorbent Assay (Merck, Germany). Some of them differentiate between Stx1 and Stx2 while others do not. The reported sensitivities and specificities of these immunoassays vary by test format and manufacturer. The standard by which each manufacturer evaluates its tests also varies; therefore, a direct comparison of performance characteristics of various immunoassays has not been performed [13].

Outbreaks of STEC infections can be contained by sanitary measures and by monitoring the water and food supply. Hence, immunization of the general population cannot be justified given the safety restrictions of a vaccine. Treatment of infected patients is needed to prevent progression of the infection to HUS [14]. However, despite remarkable advances in understanding STEC pathogenesis, the clinical options for treatment remain limited to mainly supportive strategies. The use of antimicrobials is controversial; mainly reflected by the effect of specific antimicrobial agents on phage induction and subsequent Stx gene expression and transcription [15], thereby allowing the undesirable release of verotoxin Stx, and thus being usually avoided [16,17,18].

There is no current approved treatment to combat or prevent illness from STEC, but several promising options for the future are under investigation. These options include several vaccine candidates such as Stx1 and Stx2 genetic toxoids, a plant-based Stx2 toxoid and a chimeric StxA2/StxB1 toxoid that elicits a neutralizing antibody response and provides protection against a lethal challenge of both Stx1 and Stx2 [19,20,21]. Passive immunization with monoclonal antibodies rose against Stx1 and Stx2 has been shown to be effective in animal models [22,23,24,25,26,27,28,29,30], and urtoxazumab, a humanized monoclonal antibody against Shiga-like toxin 2 is undergoing clinical trials [31].

Therefore, these compelling, important aspects of STEC infection relative to diagnosis and therapy motivated us to produce monoclonal antibodies against Stx1 and Stx2. In the present study, we demonstrated their interactions and neutralization of Shiga toxins, and the results obtained support their use as tools in STEC diagnosis and encourage future studies to investigate their protective efficacy.

## 2. Material and Methods

### 2.1. Toxins, Chemicals, Reagents, Antibodies and Supplies

Purified Stx1 and Stx2 were purchased from Tufts University School of Medicine, Boston, MA, USA. A Protein A affinity chromatography column was bought from GE Healthcare. Bovine serum albumin (BSA), polyethylene glycol 1500, goat anti-mouse IgG peroxidase-conjugated antibodies, goat anti-mouse IgG-FITC conjugated, ο-phenylenediamine (OPD) and 3’3’-diaminobenzidine (DAB) were acquired from Sigma Aldrich (St Louis, MO, USA). Horseradish peroxidase-conjugated rabbit anti-mouse-IgG+A+M purchased from Zymed (San Francisco, CA, USA). HAT—(10 mM hypoxanthine, 40 μM aminopterin and 1.6 mM thymidine) and fetal bovine serum (FBS) were acquired from GibcoBRL (Itapevi, SP, Brazil). Nitrocellulose membrane Hybond C-Extra was acquired from Amersham Biosciences (Little Chalfont, UK). For ELISA assays, we employed MaxiSorp microplates from Nunc (Rochester, NY, USA) and assay measurements were done in a Multiskan EX ELISA reader from Labsystems (Milford, MA, USA).

### 2.2. Bacterial Isolates

In this study, 45 STEC isolates from human and animal sources, belonging to different serotypes previously characterized by the presence of *stx* gene by polymerase chain reaction (PCR) [32,33,34] were used for MAbs characterization against Stx1 and Stx2. EDL933 was included in the assays as a positive control of the strain producing Stx1/Stx2. All strains were cultivated as described by Rocha and Piazza [33] to enhance expression of Stx by bacterial isolates and for Vero cell cytotoxicity assay (VCA)/neutralization assay. 

### 2.3. Stx1 and Stx2 Toxins and Toxoids

Toxins were converted to toxoids by either formaldehyde or glutaraldehyde treatment using the protocol described by Donohue-Rolfe *et al.* [35] and Brown *et al.* [36], respectively, before immunization of the mice. 

### 2.4. Anti-Stx1 and Anti-Stx2 Monoclonal Antibody (MAb) Production

Four to six week-old female BALB/c mice were immunized via the footpad with 10 µg Stx1 or 3 µg Stx2 toxoid adsorbed to 250 µg aluminum hydroxide. The immunization protocols consisted of three booster injections of the toxoid (10 µg) in 0.01 M phosphate buffered saline, pH 7.4 (PBS) at four-week intervals for Stx1 toxoid, and two booster injections (15 µg) with a 15-day interval for Stx2 toxoid. The experiments were conducted in agreement with the Ethical Principles in Animal Research, adopted by the Brazilian College of Animal Experimentation, and they were approved by the Ethical Committee for Animal Research of Butantan Institute (469/08).

The mouse with the highest antibody titer was boosted with 10 µg Stx1 or 15 µg Stx2 toxoid three days prior to cell fusion. Serum samples were obtained just before the first immunization by the retro-orbital sinus method to be used as the negative control in specific antibody evaluation. Serum samples were also obtained ten days after the last antigen injection and subsequently analyzed by ELISA.

The popliteal lymphnode cells were fused with SP2/O-Ag14 mouse myeloma cells (2:1) using polyethylene glycol 1500 [37], with modifications. Hybrids were selected in RPMI 1640 medium plus 3% HAT containing 10% FBS at 37 °C and 5% CO_2_. The supernatant fluids were screened for species-specific antibodies by indirect ELISA. 

For ELISA, hybridoma supernatant (100 µL) was added to wells of a 96-well plate previously coated with 0.1 µg-purified toxins to screen cultures for antibody production. Antibody-secreting cells were expanded and cloned twice at limiting dilution. Hybridomas secreting MAbs were selected using STEC and other non-producing Stx isolates by capture ELISA.

### 2.5. MAb Characterization

#### 2.5.1. MAb Isotyping and Purification

The microplate was coated overnight at 4 °C with 1 µg goat anti-mouse IgG1, IgG2a, IgG2b, IgG3, IgA, IgM and IgE in 0.05 M sodium carbonate-bicarbonate buffer (pH 9.6). Hybridoma supernatants were incubated with each of the isotype followed by incubation with horseradish peroxidase-conjugated rabbit anti-mouse-IgG+A+M (1:1,000). The supernatants from selected clones were filtered (0.45 µm) and purified by protein A affinity chromatography. MAb purity was determined by 15% polyacrylamide gel electrophoresis containing sodium dodecyl sulfate (SDS-PAGE) [38,39] staining with Coomassie blue R-250. 

#### 2.5.2. Interaction of MAbs with Toxin: Definition of Detection Limit, Affinity and Stability

Anti-Stx1 and Stx2 MAbs features were determined by ELISA. The detection limit was established using toxin concentrations from 100 to 0.09 ng coated on microplates in a PBS solution at 4 °C for 16–18 h. After blocking (1% BSA at 37 °C for 30 min), toxins were incubated with 0.2 µg anti-Stx1 or anti-Stx2 monoclonal antibody diluted in blocking buffer at 37 °C for 1 h, followed by incubation with goat anti-mouse peroxidase-conjugated antibody diluted 1:5,000, at 37 °C for 1 h. The reaction was developed with 0.5 mg/mL OPD plus hydrogen peroxide (H_2_O_2_), and stopped by the addition of 1 N HCl. The absorbance was measured at 492 nm in a Multiskan EX ELISA reader.

The cross reactivity between MAbs and toxins was determined by indirect ELISA using different anti-Stx1 or anti-Stx2 MAb concentrations (ranged from 1.0 pg to 10 μg) with a predetermined toxin concentration (0.1 μg) as antigen.

Three-step ELISA described by Friguet *et al.* [40] was employed to determine the dissociation constants (*K_D_*) of antigen-antibody interactions under equilibrium conditions. Briefly, dilutions of different MAbs, selected in the linear part of the ELISA titration curves (determined by linear regression) were incubated overnight at 4 °C with various concentrations of antigen. The concentration of free MAb was determined by ELISA, where aliquots (100 μL) of incubation medium were transferred to the wells of microtiter plates previously coated with Stx1 or Stx2. Dissociation constants were deduced from Scatchard plots.

The stability of anti-Stx1 and Stx2 MAbs was determined by indirect ELISA in which MAb solutions were kept at room temperature, or at 37 °C, 50 °C, 60 °C, 80 °C, 90 °C and 100 °C for 10 min. Tests were performed at different times up to 2 h.

#### 2.5.3. Stx1 and Stx2 Characterization by Immunoblotting

The reactivity of antibodies to purified Stx1 and Stx2 toxins was tested by immunoblotting using the monoclonal antibodies. Briefly, 10 μg per slot of toxins were submitted to 15% SDS-PAGE. After electrophoresis, the separated proteins were transferred to a nitrocellulose membrane at 150 mA for 18 h at 4 °C. The membrane was blocked with 1% BSA for 2 h and incubated with anti-Stx1 or anti-Stx2 monoclonal antibodies (50 µg) at room temperature for 2 h and at 4 °C for 18 h. Next, the membrane was washed and incubated at room temperature for 1 h with peroxidase-conjugated goat anti-mouse IgG (1:5,000). After washing, DAB plus H_2_O_2_ were added and the reaction was stopped after 15 min by the addition of distilled water.

#### 2.5.4. MAb Reactivity to Shiga Toxin-Producing *E. coli*

MAb reactivity to the toxins expressed by STEC isolates was determined by capture ELISA using microplates coated with IgG-enriched fraction of anti-Stx1 (1 µg) or anti-Stx2 (3 µg) from rabbit polyclonal serum [35,36] at 4 °C for 16–18 h. After blocking with 1% BSA at 37 °C for 30 min, 100 µL of isolates supernatants were incubated at 37 °C for 2 h followed by incubation with either 0.5 µg MAb anti-Stx1 or 0.5 µg MAb anti-Stx2. Antigen-antibody binding was detected by the addition of goat anti-mouse IgG-peroxidase conjugate (1:5,000) and OPD (0.5 mg/mL) and H_2_O_2_ as enzyme substrates, and the peroxidase reaction was stopped by the addition of 1 N HCl. The absorbance was measured at 492 nm in a Multiskan EX ELISA reader. MAb reactivity to Stx expressed by STEC isolates was arbitrarily defined as low (1–30 ng), medium (31–60 ng) and high (61–100 ng) compared to the absorbance obtained with the reactivity of 100 ng of purified toxins, which we considered a high reactivity level.

### 2.6. Vero Cell Toxin Assays

Vero cells (1 × 10^5^ cells/mL) were grown in 96-well plates in Dulbecco’s medium (DMEM) in the presence of 10% FBS for 24 h for Vero cell assay (VCA) and neutralization assays. Cells were also cultivated under the same conditions in 24-well plates containing 13 cm diameter coverslips for immunofluorescence assay.

#### 2.6.1. VCA

Half-maximal cytotoxic doses (CD_50_) of the toxins were determined by VCA. Log_10_ dilutions from 0.001 pg to 10 µg of each toxin were incubated with Vero cells at 37 °C, in a 5% CO_2_ atmosphere for 72 h. This activity was determined as described by Gentry and Dalrymple [41]. Cell viability was determined using a spectrophotometer after staining the cells with crystal violet, and the percentage of cytotoxicity was calculated using the formula: control A_595_ nm minus sample A_595_ nm, divided by control A_595_ nm. Cell monolayer in presence of medium and without toxins was employed as control of cell viability. These assays were performed twice in duplicate.

#### 2.6.2. Monoclonal Antibody Neutralizing Assays

Neutralizing ability of anti-Stx1 or anti-Stx2 monoclonal antibodies was determined by incubating each toxin at the CD_50_ with different MAb concentrations from 0.4 pg to 100 µg for anti-Stx1 or 0.4 pg to 500 µg for anti-Stx2. Culture supernatants of STEC producing Stx1 and/or Stx2 were incubated at 1:10 dilution with 30 μg anti-Stx1 and 500 μg anti-Stx2 monoclonal antibodies at 37 °C for 2 h. Stx1- and Stx2-specific rabbit antisera were employed as neutralizing activity controls [33,34]. After incubation, these mixtures were tested as described by Beutin *et al.* [42]. These assays were performed three times in duplicate.

#### 2.6.3. Immunofluorescence and Confocal Analysis

The immunofluorescence assay was performed as described by Dorsey *et al.* [43]. Toxins and cells were incubated at 37 °C for 0, 2, 6 and 24 h using 0.1 pg Stx1 or 640 pg Stx2. After this period, cells were washed with DMEM containing 2% FBS and then fixed with 4% ρ-formaldehyde for 16–18 h at 4 °C. The cells were washed three times with PBS and 1% glycine in PBS was added to quench excess aldehyde groups. After blocking with PBS containing 1% BSA and incubation with 100 µg anti-Stx1 or 200 µg anti-Stx2 diluted in blocking buffer at 37 °C for 1 h, the antigen-antibody reaction was detected by the addition of anti-mouse IgG-FITC (1:100). The reaction was visualized with a confocal laser-scanning microscope (LSM 510 META). The image was obtained and analyzed with a confocal microscope Zeiss LSM image browser, and the cut-off was defined as the control reaction of Vero cells with either MAb anti-Stx1 or anti-Stx2 plus anti-mouse IgG-FITC in the absence of toxin. 

#### 2.6.4. Statistical Analysis

The differences between isolates with regard to cytotoxicity and their corresponding neutralization by the MAbs was analyzed by GraphPrism^®^ 5.01, using Student’s *t*-test and two-away ANOVA. The differences were considered statistically significant when *p* ≤ 0.05.

## 3. Results

### 3.1. Interaction of Monoclonal Antibodies with Stx1 and Stx2

The detoxification process was employed to reduce Stx1 and Stx2 toxicity to less than 1% for mouse immunization. The immunization protocols used for Stx1 or Stx2 in BALB/c mice generated high IgG antibody titers for both toxins. The mean optical density at 492 nm was 1.0 up to 3200-fold serum dilution in mice immunized with the two toxins.

**Figure 1:**
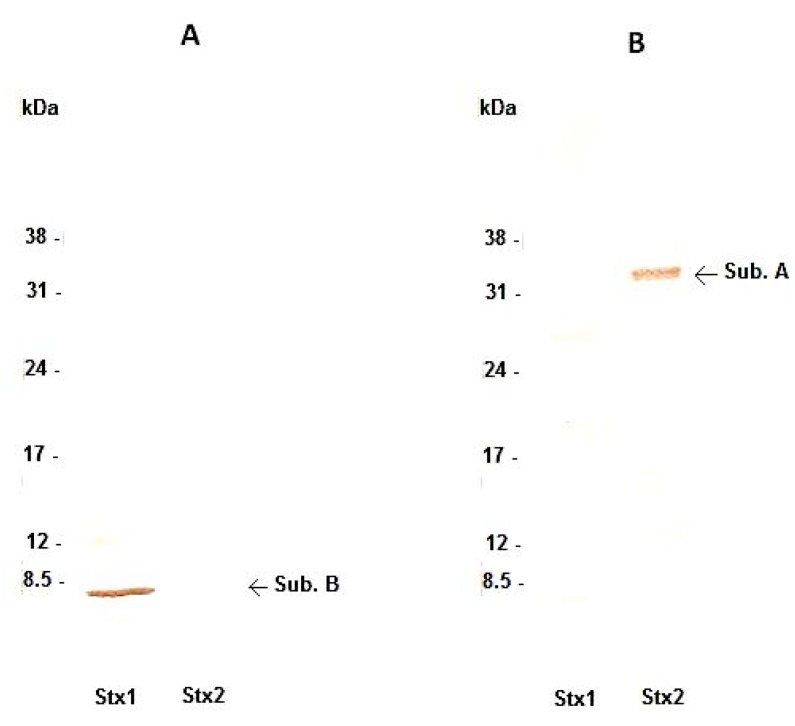
Nitrocellulose membranes containing purified toxins Shiga toxins (Stx)1 and Stx2. Immunoblotting reaction was carried out using anti-Stx1 MAb (A) and anti-Stx2 MAb (B). Apparent molecular weights found showed that the MAbs recognized their corresponding toxin. Arrows indicate toxin subunits.

Secretory hybridomas of antibodies against Stx1 and Stx2 were obtained and subcloned by limiting dilution. Anti-Stx1 and anti-Stx2 MAbs produced by the selected clones (3E2 and 2E11, respectively), were classified as IgG1 and showed reactivity with their respective toxins by immunoblotting: anti-Stx1 MAb bound to the B subunit (Figure 1A) with a dissociation constant of 2.5 × 10^−10^ M (Table 1), while anti-Stx2 MAb bound to the A subunit (Figure 1B) with a dissociation constant of 6.1 × 10^−10^ M (Table 1).

**Figure 2:**
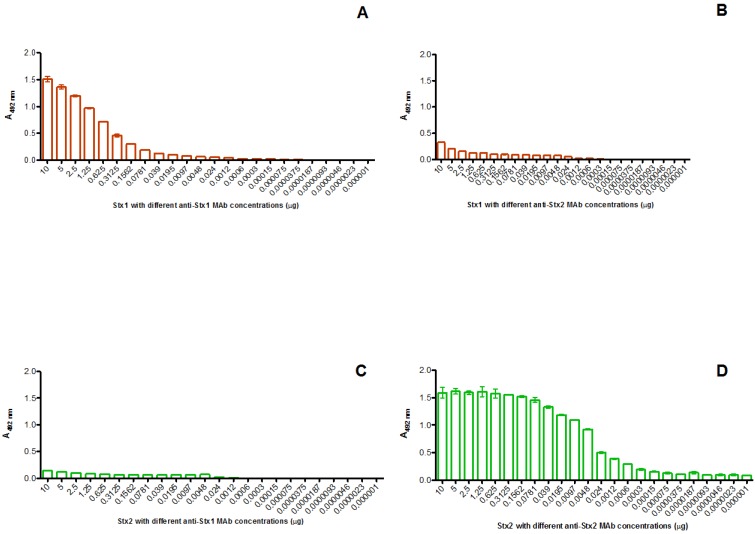
Indirect ELISA using MAbs 3E2 and 2E11 and purified toxins Stx1 or Stx2. ELISA microtiter was coated with 0.1 μg of Stx1 (A and B) and Stx2 (C and D). The reaction was carried out using different concentrations of anti-Stx1 MAb, 3E2 (A and C) or anti-Stx2 MAb 2E11 (B and D). The cut-off was defined as 0.1 OD. Cross reactivity occurred at high MAbs concentration.

Using 200 ng MAbs to determine their ability to bind to the respective toxin, the detection limit was 6.2 ng for anti-Stx1 and 12.5 ng for anti-Stx2, using an optical density of 0.1 as cut-off (Table 1). The equivalent cut-off was used for determining the reactivity and cross reactivity of MAbs and toxins. Anti-Stx1 (3E2) reacted with 0.1 μg of Stx1 up to 19.5 ng (Figure 2A), 2.5 μg of anti-Stx1 MAb was necessary to detect the same amount of Stx2 (Figure 2B). For anti-Stx2 (2E11) the reactivity with the Stx2 was up to 18.7 pg (Figure 2D) and 0.31 μg was necessary to detect Stx1 (Figure 2C). These results show that cross reactivity occurrs only in the presence of higher MAb concentrations, which are excessive to detected theirs respective toxins.

The MAb stability parameters were quite different for both toxins. Anti-Stx2 MAb was stable up to 70 °C, while anti-Stx1 lost stability at 50 °C (Table 1). Moreover, a higher temperature and more time were necessary for anti-Stx2 MAb to lose immunoreactivity compared with anti-Stx1 MAb (Table 1).

**Table 1:** Features of anti-Stx1 and anti-Stx2 MAbs.

MAb characteristic	Anti-Stx1	Anti-Stx2
Hybridomas	3E2	2E11
Dissociation constant (*K_D_*)	2.5 × 10^−10^ M	6.1 × 10^−10^ M
Detection limit (200 ng)	6.2 ng	12.5 ng
Thermostability	50º C	70º C
Total loss of immunoreactivity (80 ºC)	1 min	5 min
Partial loss of immunoreactivity	60 ºC and 70 ºC	80 ºC and 90 ºC.

**Figure 3:**
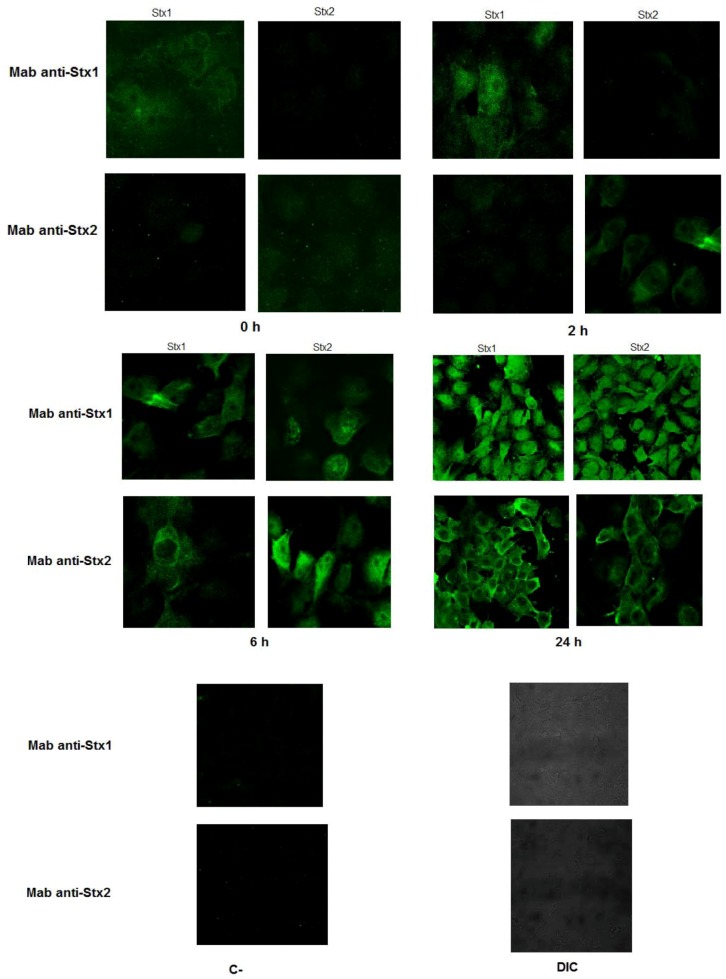
Immunofluorescence assay after Stx1 or Stx2 interaction with Vero cells for 0, 2, 6 and 24 h. The reaction was carried out by incubating the cells with anti-Stx1 or anti-Stx2 MAbs. Cells labeled with FITC displayed an apple green fluorescence, showing that MAbs were able to recognize the toxins after cell interaction. Reactivity of anti-Stx1 or anti-Stx2 MAbs with cells and the anti-IgG mouse FITC in the absence of toxins was used as the negative control (C-), besides the differential interference contrast (DIC) of negative control. Reactivity was visualized with a confocal laser-scanning microscope (LSM 510 META).

The kinetics of the toxins was analyzed by incubating the toxins at 0, 2, 6 and 24 h with Vero cells. After fixation, these interactions were visualized after the MAb reaction (Figure 3). In the early periods (0 and 2 h) each MAb recognized only their respective toxins, but after 6 h both MAbs were able to identify the presence of both toxins in Vero cells, shown by immunofluorescence. Using confocal microscopy, we observed that fluorescence emission with the homologous toxin was more intense. Presence of either Stx1 or Stx2 was observed along the cell when anti-Stx1 was employed (Figure 3). The pattern of recognition by the anti-Stx2 MAbs with either Stx1 or Stx2 was limited to the cell border (Figure 3). Assay specificity was assured by interaction of monoclonal antibodies with Vero cells in the absence of toxin (Figure 3).

MAb reactivity with STEC isolates was determined by capture ELISA. Data from this experiment showed not only MAb reactivity but also allowed us to infer which toxins were produced by STEC isolates, thus anti-Stx1 and anti-Stx2 MAbs detected the toxins expressed even at low levels (Table 2), demonstrating the diagnostic sensitivity of the MAbs. Furthermore, the reactivity of the MAbs matched the presence of the *stx1* and *stx2* genes, confirming their specificity. Just in one case (isolate O36, O75:H8), *stx2* gene was amplified but the respective protein was not detected by anti-Stx2 MAb. However, *stx1* gene was detected in the same isolate, and anti-Stx1 MAb recognized the respective toxin.

**Table 2:** Shiga toxin (Stx)-producing *Escherichia coli* characteristics.

Strain number	Serotype	Gene presence	Reactivity to MAbs	
			Anti-Stx1 Anti-Stx2	
597	OR:NM	*stx1*	H	-
1132	ONT:H49	*stx2*	-	H
1189	ONT:H49	*stx2*	-	H
3003	O48:H7	*stx1/stx2*	M	M
4123	O26:H11	*stx1*	M	-
D360/4/1	O26:H11	*stx1*	M	-
1557-77	O26:H11	*stx1*	H	-
H30	O26:H11	*stx1*	M	-
H19	O26:H11	*stx1*	M	-
EPEC199	O26:H11	*stx1*	M	-
3529	O26:H11	*stx1*	M	-
82	O157:H7	*stx1*	H	-
46240	O157:H7	*stx1/stx2*	M	H
3104-88	O157:H7	*stx1/stx2*	M	H
3077-88	O157:H7	*stx1*	M	-
C7-88	O157:H7	*stx1*	L	-
C1520-77	O157:H7	*stx1/stx2*	H	H
1	O157:H7	*stx2*	-	H
2	O157:H7	*stx2*	-	H
4	O93:H19	*stx1/stx2*	H	L
5	O55:H19	*stx1*	L	-
9	O103:H2	*stx1/stx2*	H	H
11	O118:H16	*stx1*	M	-
16	O26:H11	*stx1*	L	-
20	O111:H8	*stx1*	L	-
23	O111:H8	*stx1*	L	-
26	O111:NM	*stx1*	M	-
27	O111:NM	*stx1*	M	-
41	ONT:NM	*stx2*	-	M
44	O98:H4	*stx1/stx2*	M	H
45	O181:H4	*stx1/stx2*	M	H
53	O98:H17	*stx1/stx2*	L	H
55	O98:H17	*stx1/stx2*	M	H
59	ONT:H16	*stx2*	-	L
66	O105:H18	*stx1/stx2*	H	H
79	O22:H16	*stx2*	-	H
81	ONT:H38	*stx1/stx2*	H	H
82	O112:H21	*stx2*	-	H
96	O93:H19	*stx2*	-	M
O1	ONT:H8	*stx1*	L	-
O17	O112:H2	*stx1*	L	-
O3	O172:NM	*stx2*	-	H
O22	ONT:H16	*stx2*	-	L
O36	O75:H8	*stx1/stx2*	M	-
O55	O146:H21	*stx1/stx2*	H	L
EDL 933	O157:H7	*stx1/stx2*	H	H

- —MAb reactivity lower than 0.99 ng; L—low level of MAb reactivity (1–30 ng); M—medium level of MAb reactivity (31–60 ng); H—high level of MAb reactivity (61–100 ng). Arbitrary classification based on the obtained absorbance with 100 ng purified Stx1 or Stx2, which was considered to be a high level of reactivity by capture ELISA.

### 3.2. Neutralizing Ability of Monoclonal Antibodies

**Figure 4:**
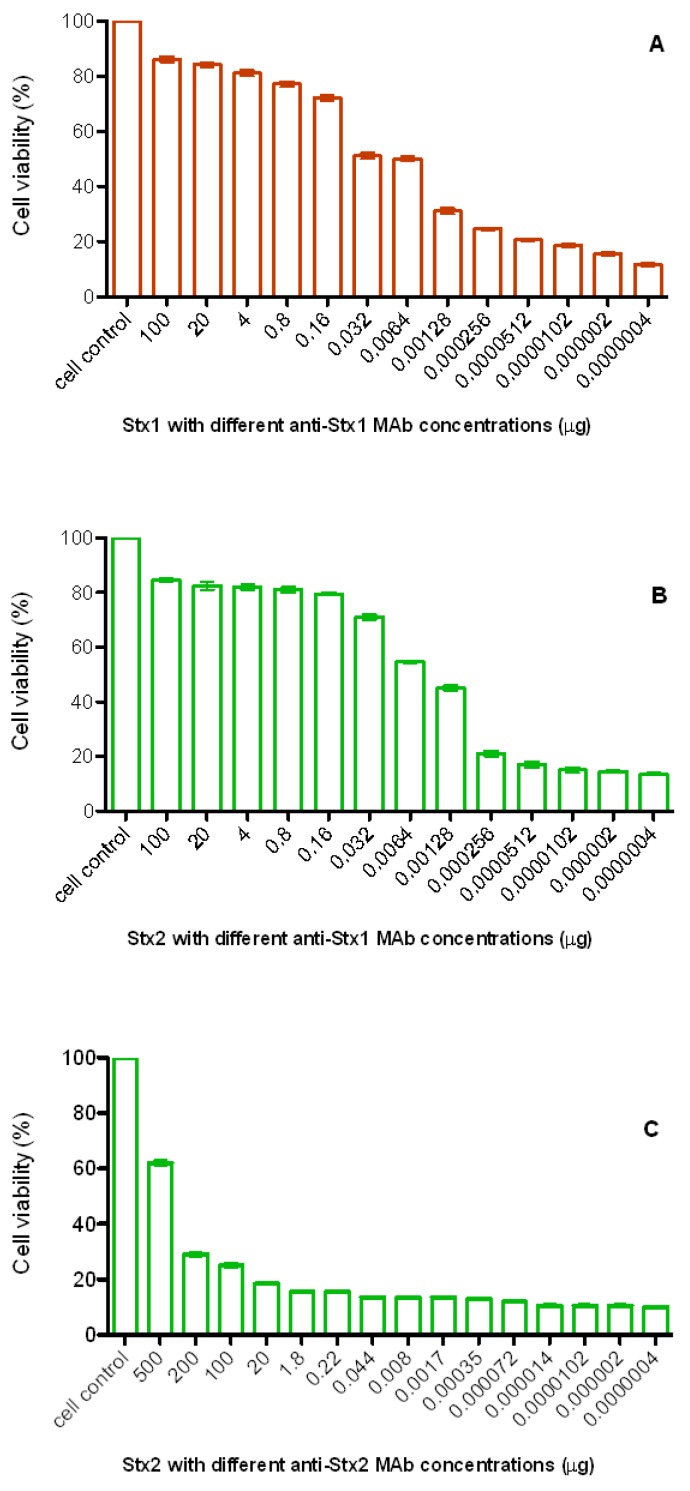
A and B—anti-Stx1 MAb showed cross-reactivity and was able to inhibit between 70 and 80% of both toxins ((A) Stx1 and (B) Stx2). Higher concentrations of anti-Stx2 MAb (100 to 500 µg) were necessary to neutralize Stx2 activity (C), but not Stx1 (data not shown). Bars represent the mean and the standard errors of the percentage of duplicates of three independent experiments.

The CD_50_ of each toxin was determined prior to neutralization assays using anti-Stx1 and anti-Stx2 MAbs. CD_50_ was defined as 10 ng and 500 ng for Stx1 and Stx2, respectively. Concentrations of anti-Stx1 MAb ranging from 1.28 ng to 100 µg neutralized between 30 and 80% of Stx1 cytotoxic activity (Figure 4A). Anti-Stx1 MAb showed cross-reactivity (Figure 4B), *i.e.*, it inhibited both Stx1 and Stx2 activity to the same extent. On the other hand, 100 µg or 200 µg anti-Stx2 MAb were necessary to neutralize ca. 35% of Stx2 activity, and 60% of neutralization was only achieved using 500 µg MAb (Figure 4C). No apparent cell damage was observed in any MAb concentration. 

**Figure 5:**
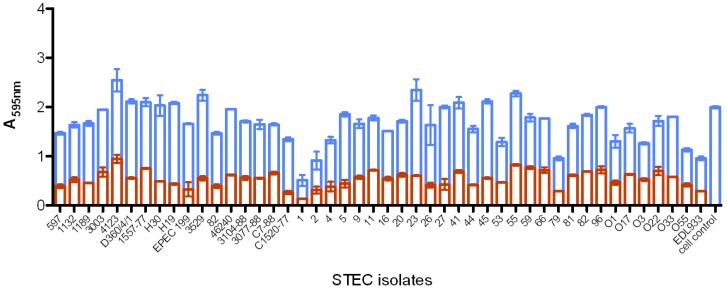
Vero cell cytotoxicity assay (VCA) and neutralization assays with culture supernatant of 46 shiga toxin-producing *Escherichia coli* (STEC) isolates producing Stx1 and/or Stx2 incubated with (blue) or without (red) anti-Stx1 and anti-Stx2 MAbs followed by cell incubation. Cytotoxicity and neutralization were determined after staining the cells with crystal violet and measuring absorbance at 595 nm. Means and variances were significantly different (*p* < 0.0001) by Student’s *t-*test and 2-way ANOVA comparing cytotoxicity and neutralization groups. Bars represent the OD means and standard errors median of duplicates of three independent experiments. A high OD value means high cell viability.

After determining the neutralization point for both MAbs, their ability to neutralize the cytotoxic activity of the STEC strains, belonging to several serotypes and showing either *stx1*, *stx2* or *stx1*/*stx2* genes, was investigated. We observed that Stx activity was neutralized (from 25 to 80%) by the MAbs in all isolates (Figure 5). Besides, using both MAbs, we were able to neutralize Stx1 and Stx2 expressed by isolates. Cellular integrity was maintained at these MAb concentrations in the absence of toxins. Means and variances were significantly different (*p* < 0.0001) by Student’s *t*-test and 2-way ANOVA, comparing the cytotoxicity and neutralization groups (Figure 5).

## 4. Discussion

A number of anti-Stx MAbs showing different features have been described in the literature [19,23,24,30,35,44,45,46,47,48,49], including their use for diagnosis, but just for Stx1 and for O157:H7 recognition [49]. Thus, raising monoclonal antibodies takes a remarkable effort, and developing them as tools for diagnosis or therapy is a major struggle. 

Anti-Stx1 and anti-Stx2 MAbs obtained in the present study were produced against toxins converted to toxoids by either formaldehyde or glutaraldehyde treatment [35,36]. The 3E2 clone was obtained in the first fusion after Stx1 treatment by glutaraldehyde and using the immunization and fusion protocols already described by our group either for the MAbs IgG2b anti-LT or IgG2b anti-intimin [50,51,52]. In contrast, the 2E11 clone was merely obtained after ten fusions, through different tested protocols either for toxoid conversion or route and schedule immunization. Among the six clones obtained, only 2E11 produced antibodies that were reactive to Stx-producing strains with high affinity and showed no reactivity with non-Stx-expressing strains (data not shown).

Anti-Stx1 and anti-Stx2 were classified as IgG1, as in case of MAbs already produced by other research groups as a consequence of the long schedule of mouse immunization with Stx toxoids [23,24,45]. Furthermore, these MAbs showed an efficient interaction with their respective toxin as indicated by their high dissociation constants and their ability to detect low levels of both purified toxins as well as low-producer isolates. In fact, among the described MAbs the affinity constant was cited in few studies. The studies by Tanikawa *et al.* [53] and Kimura *et al.* [26] showed the *K_D_* of 2.6 × 10^−10^ M for anti-Stx1 MAb and the *K_D_* of 2.3 × 10^−9^ M for anti-Stx2A MAb, which are similar to our described MAbs.

Another important characteristic was the stability of MAbs at high temperatures and the reactivity of anti-Stx1 or anti-Stx2 with their homologous toxin by indirect ELISA. 3E2 reacted with the B subunit of Stx1, while 2E11 recognized the A subunit of Stx2 by immunoblotting. This variation in reactivity is very common and has been shown by other groups with anti-Stx1 MAbs that either react with the A subunit [28] or the B subunit [24] or are conformational [30]. MAbs against Stx2 reacting with either the A or B subunit have also been described [23,44,45,47]. In the present study, both MAbs recognized only their specific denatured toxin by immunoblotting. Besides, the cross reactivity was only observed using high antibody concentrations by indirect ELISA. Surprisingly, when native protein was used by *in vitro* interaction assay, both MAbs showed cross reactivity after 6 h of toxin-Vero cells interaction, despite different recognition patterns.

An *in vitro* cytotoxicity assay was performed to evaluate the conditions by which the MAbs were able to neutralize the cytotoxic effects of Stx1 and/or Stx2. The neutralizing ability of the produced MAbs showed that anti-Stx1 MAb neutralized the binding of Stx1 and Stx2 to Vero cells. On the other hand, anti-Stx2 neutralized only the homologous toxin, and a higher concentration of this MAb was necessary. The correlation between subunit reactivity and neutralizing ability is variable. For example, Smith *et al.* [30] characterized a conformational MAb that neutralizes the Stx2 B subunit. The opposite was observed by Sheoran *et al.* [47] since the two MAbs tested failed to neutralize Stx2c *in vitro* due to their stronger reactivity with the B subunit than with the A subunit of Stx2 by immunoblotting.

The clinical options for treatment of STEC still remain limited to mainly supportive strategies despite researchers’ efforts in understanding this pathogen. No MAb is currently approved for clinical use, but promising options for the future are under investigation, including urtoxazumab against Stx2, which is undergoing clinical trials and appears to be safe, making it a potential candidate for the prevention of HUS in pediatric patients [31]. Also, another human monoclonal antibody [27,28] protected mice against lethal challenges with Stx2 and Stx2 variants [47]. Preclinical evaluation in a piglet model of infection showed protection against Stx2-induced fatal neurological symptoms, even when the antibody was administered after the onset of diarrhea and oral STEC challenge [54].

Given that STEC can produce any combination of Stx1, Stx2, and/or variants [55], an ideal therapeutic formulation should include MAbs specific for all them, which could provide broad-spectrum protection against Stx1 and/or Stx2 [27,28,47]. Our results suggest that the manipulation by site-directed mutagenesis of the single chain fragment variable (ScFv) of MAbs should be interesting to improve their affinity and allow the large-scale production of recombinant antibodies with desirable sensitivity and specificity. This is currently under way in our laboratory.

Moreover, the previously described IgG-enriched fraction of anti-Stx1 or anti-Stx2 from rabbit polyclonal antiserum [33,34] together with anti-Stx1 or anti-Stx2 MAbs, showed high sensitivity in detecting Stx even in low-producer isolates by capture ELISA. Therefore, both MAbs can be used as tools for the diagnosis of STEC in view of their described features. Since antibody neutralizing efficiency *in vivo* usually correlates with its ability to protect cells against Stx-mediated toxicity [56], the *in vitro* neutralizing abilities of the described MAbs against the Shiga toxins encourage future studies to investigate their protective efficacy.
